# Supplementary material for: Evaluation of Antibacterial Activity against Nosocomial Pathogens of an Enzymatically Derived α-Aminophosphonates Possessing Coumarin Scaffold
Source: Int J Mol Sci. 2023 Oct 4;24(19):14886. doi: 10.3390/ijms241914886 (PMC10574003; doi:10.3390/ijms241914886)
Supplement: Supplementary file 1 [file ijms-24-14886-s001.zip › ijms-2635160-supplementary.pdf]

Supporting Information

Evaluation of Antibacterial Activity against Nosocomial Pathogens of an Enzymatically Derived α-Aminophosphonates Possessing Coumarin Scaffold

Paweł Kowalczyk [1,*], Dominik Koszelewski [2,*], Anna Brodzka [2], Karol Kramkowski [3] and Ryszard Ostaszewski [2]

1 Department of Animal Nutrition, The Kielanowski Institute of Animal Physiology and Nutrition, Polish Academy of Sciences, Instytucka 3, 05-110 Jabłonna, Poland
2 Institute of Organic Chemistry, Polish Academy of Sciences, Kasprzaka 44/52, 01-224 Warsaw, Poland; anna.brodzka@icho.edu.pl (A.B.); ryszard.ostaszewski@icho.edu.pl (R.O.)
3 Department of Physical Chemistry, Medical University of Bialystok, Kili'nskiego 1 Str., 15-089 Białystok, Poland; kkramk@wp.pl
* Correspondence: p.kowalczyk@ifzz.pl (P.K.); dominik.koszelewski@icho.edu.pl (D.K.); Tel.: +48-227653301 (P.K.); +48-223432012 (D.K.)

Dimethyl ((phenyl)((2-oxo-4-(trifluoromethyl)-2H-chromen-7-yl)amino)methyl) phosphonate (**1**): ^{1}H NMR (400 MHz, $CDCl_3$) δ 7.45 (ddd, *J* = 8.9, 6.0, 2.1 Hz, 3H), 7.40–7.29 (m, 3H), 6.67 (dd, *J* = 8.9, 2.4 Hz, 1H), 6.44 (dd, *J* = 10.2, 1.6 Hz, 2H), 5.85 (t, *J* = 8.8 Hz, 1H), 4.80 (dd, *J* = 24.2, 7.7 Hz, 1H), 3.80 (d, *J* = 10.9 Hz, 3H), 3.44 (d, *J* = 10.6 Hz, 3H); ^{13}C NMR (100 MHz, $CDCl_3$) δ 158.9, 156.6, 150.4, 143.2, 141.6, 134.1, 129.1, 129.0, 127.6, 124.3, 112.3, 99.7, 55.9, 54.3, 53.7, 53.7.

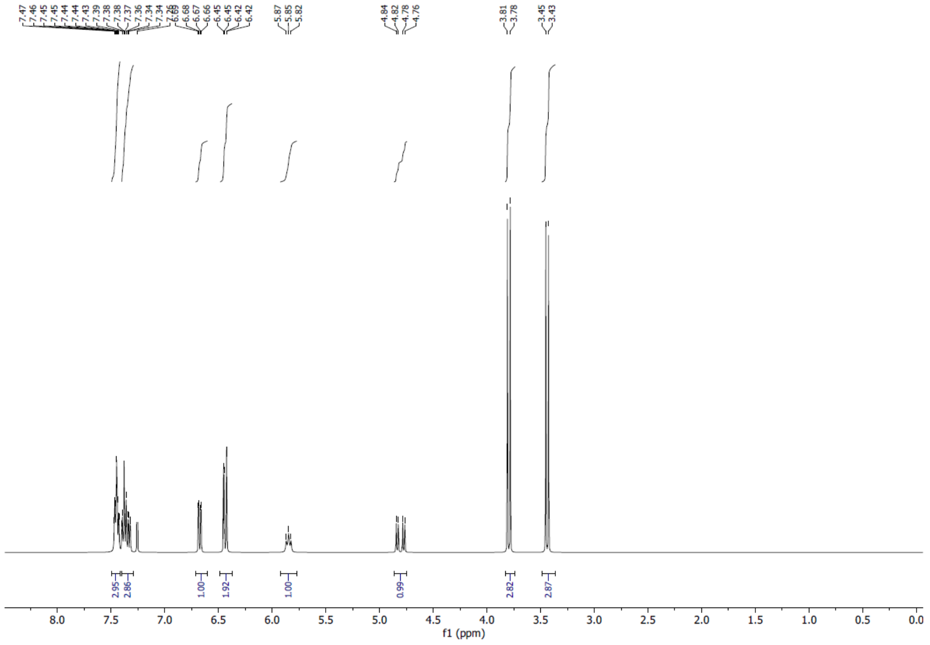

Figure S1. 1H NMR (400 MHz, CDCl**3**) spectra of compound **1**

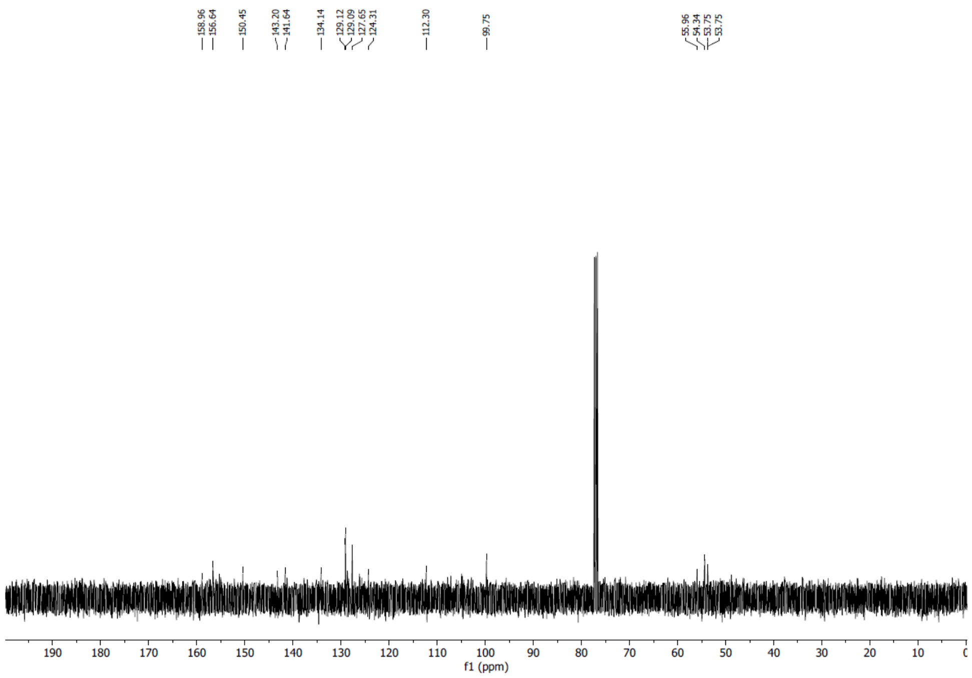

Figure S2. ^{13}H NMR (100 MHz, $CDCl_3$) spectra of compound **1**

Dimethyl ((4-methoxyphenyl)((2-oxo-4-(trifluoromethyl)-2H-chromen-7-yl)amino) methyl)phosphonate (2). ^{1}H NMR (400 MHz, $CHCl_3$) δ 7.44–7.40 (m, 1H), 7.37 (dd, *J* = 8.8, 2.3 Hz, 2H), 6.89 (d, *J* = 8.4 Hz, 2H), 6.64 (dd, *J* = 8.9, 2.4 Hz, 1H), 6.43 (d, *J* = 8.4 Hz, 2H), 5.76 (t, *J* = 8.6 Hz, 1H), 4.75 (dd, *J* = 23.7, 7.6 Hz, 1H), 3.79 (s, 3H), 3.77 (d, *J* = 10.6 Hz, 3H), 3.46 (d, *J* = 10.6 Hz, 3H); ^{13}C NMR (100 MHz, $CDCl_3$) δ 159.9, 156.6, 150.6, 150.5, 128.9, 128.8, 126.1, 125.8, 125.8, 114.6, 114.5, 112.3, 104.8, 99.7, 55.3, 54.2, 53.7, 53.6, 52.0.

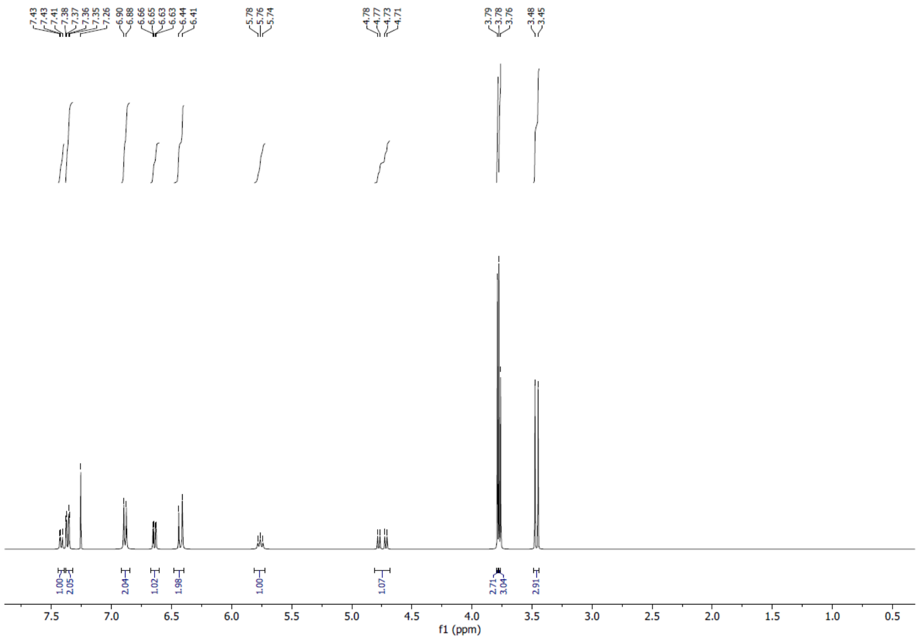

Figure S3. ^{1}H NMR (400 MHz, $CDCl_3$) spectra of compound **2**

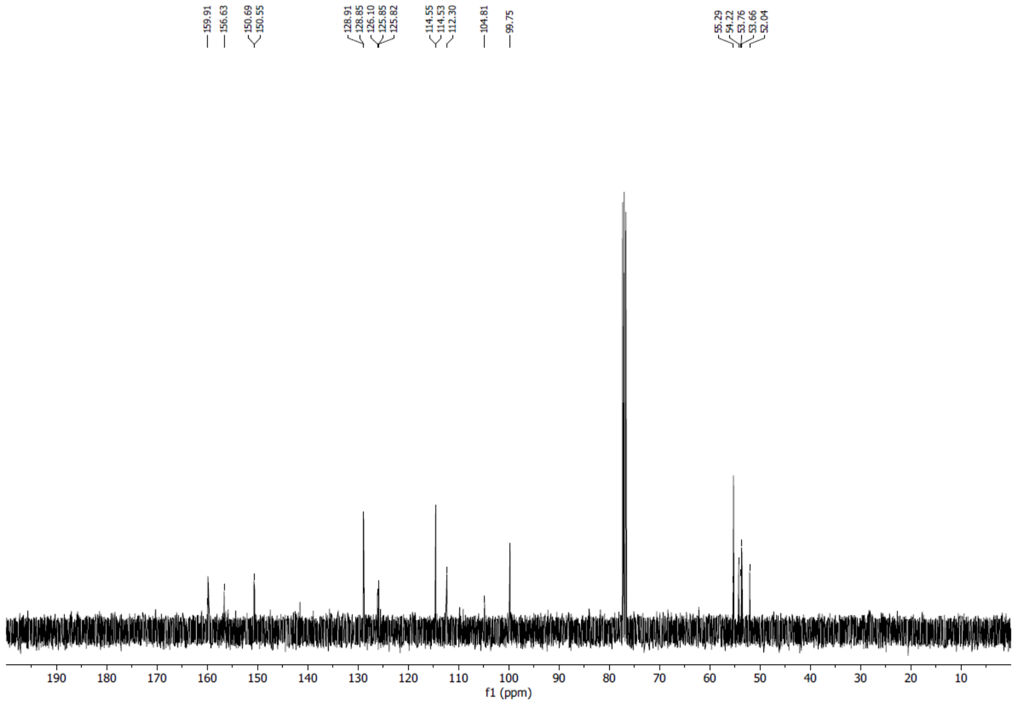

Figure S4. ^{13}H NMR (100 MHz, $CDCl_3$) spectra of compound **2**

Dimethyl ((4-methylphenyl)((2-oxo-4-(trifluoromethyl)-2H-chromen-7-yl)amino) methyl)phosphonate (3). ^{1}H NMR (400 MHz, $CDCl_3$) δ 7.43 (dd, *J* = 8.9, 2.0 Hz, 1H), 7.33 (dd, *J* = 8.2, 2.3 Hz, 2H), 7.17 (d, *J* = 8.0 Hz, 2H), 6.64 (dd, *J* = 8.9, 2.4 Hz, 1H), 6.43 (d, *J* = 8.0 Hz, 2H), 5.66 (t, *J* = 8.8 Hz, 1H), 4.76 (dd, *J* = 23.9, 7.6 Hz, 1H), 3.78 (d, *J* = 10.8 Hz, 3H), 3.46 (d, *J* = 10.6 Hz, 3H), 2.32 (s, 3H); ^{13}C NMR (100 MHz, $CDCl_3$) δ 159.9, 156.6, 150.8, 150.7, 138.5, 131.1, 131.0, 129.8, 129.7, 127.7, 127.6, 126.0, 112.3, 109.6, 109.5, 104.7, 99.7, 55.6, 54.3, 54.2, 53.7, 52.0, 21.1.

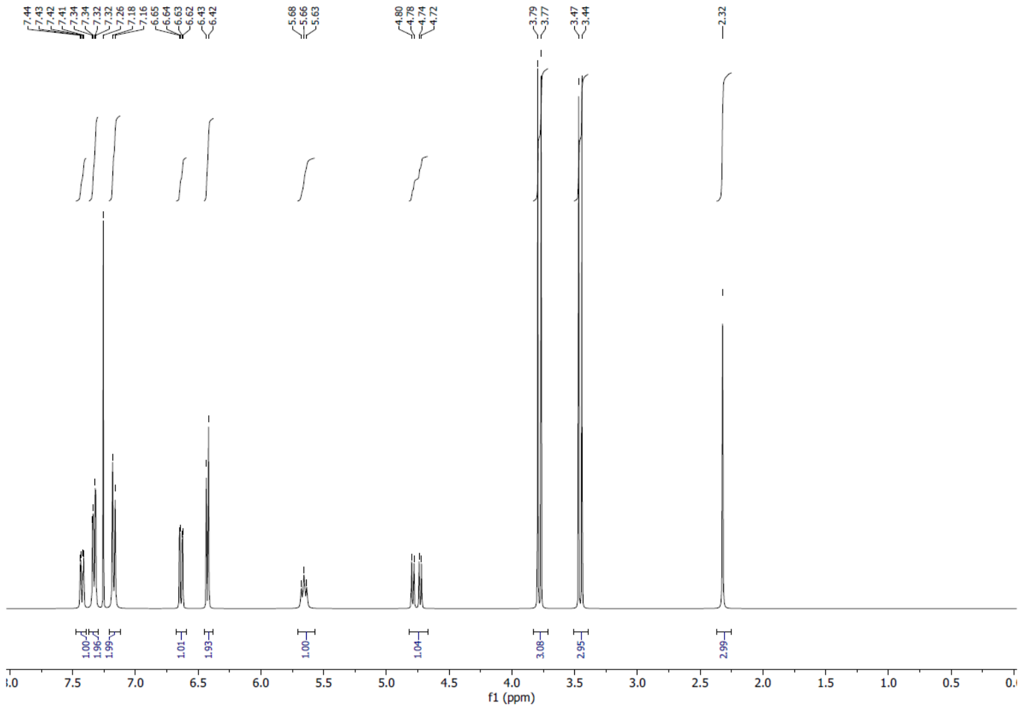

Figure S5. ^{1}H NMR (400 MHz, $CDCl_3$) spectra of compound **3**

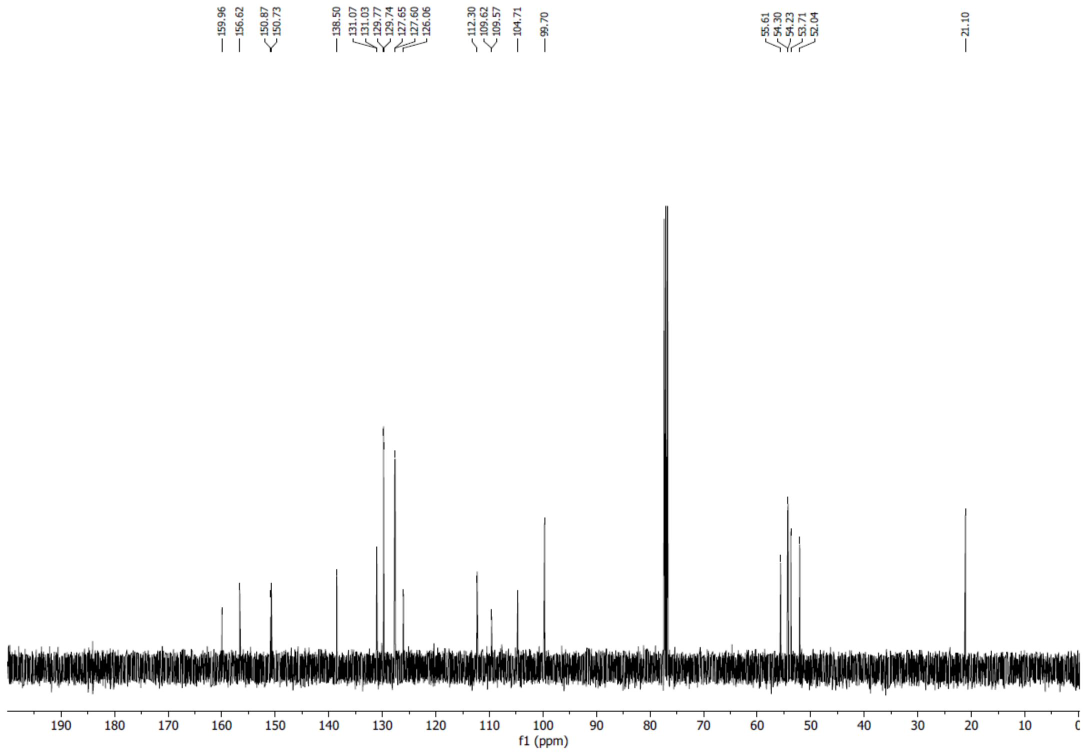

Figure S6. ^{13}H NMR (100 MHz, $CDCl_3$) spectra of compound **3**

Dimethyl ((4-fluorophenyl)((2-oxo-4-(trifluoromethyl)-2H-chromen-7-yl)amino) methyl)phosphonate (4). ^{1}H NMR (400 MHz, $CDCl_3$) δ 7.44 (ddt, *J* = 8.8, 5.5, 2.1 Hz, 3H), 7.12–6.99 (m, 2H), 6.65 (dd, *J* = 8.9, 2.4 Hz, 1H), 6.48–6.37 (m, 2H), 5.94 (t, *J* = 8.8 Hz, 1H), 4.80 (dd, *J* = 24.1, 7.7 Hz, 1H), 3.79 (d, *J* = 10.8 Hz, 3H), 3.51 (d, *J* = 10.6 Hz, 3H); ^{13}C NMR (100 MHz, $CDCl_3$) δ 159.8, 156.6, 150.5, 150.3, 130.0, 129.5,129.4, 129.3, 126.2, 116.3, 116.2, 116.1, 116.0, 112.1, 110.0, 104.9, 99.7, 60.3, 54.2, 54.1, 53.8, 53.7.

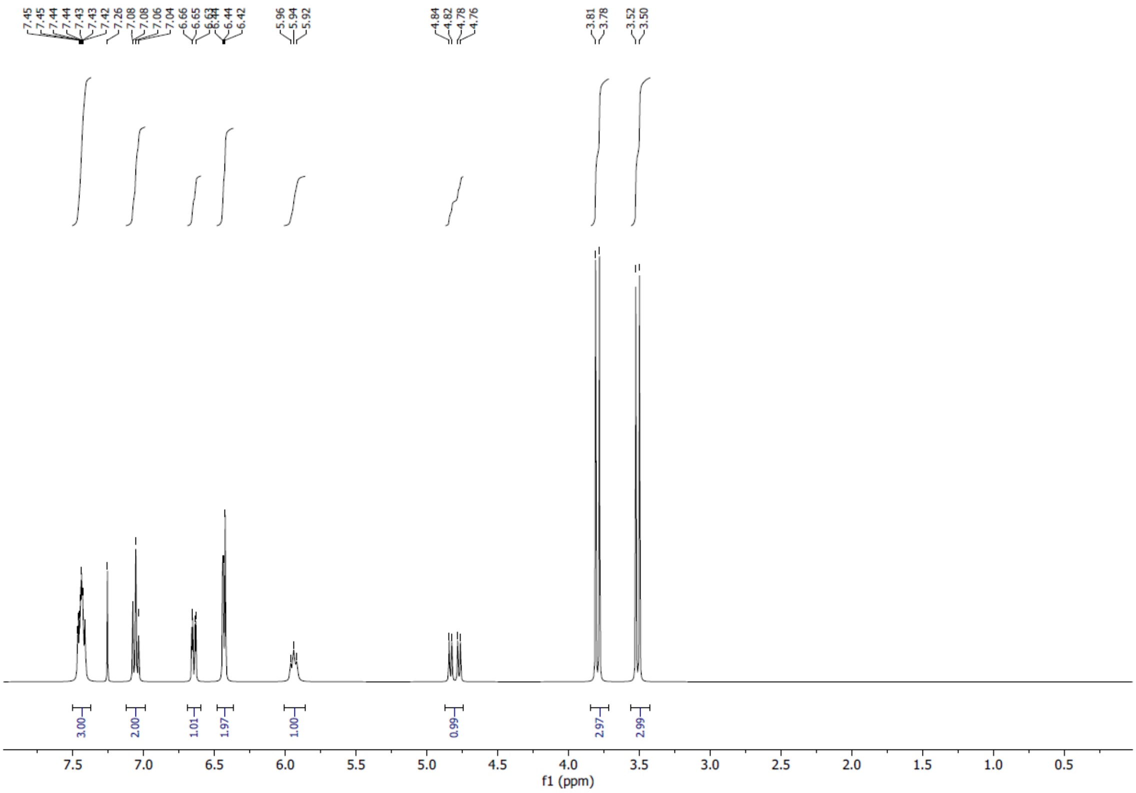

Figure S7. ^{1}H NMR (400 MHz, $CDCl_3$) spectra of compound **4**

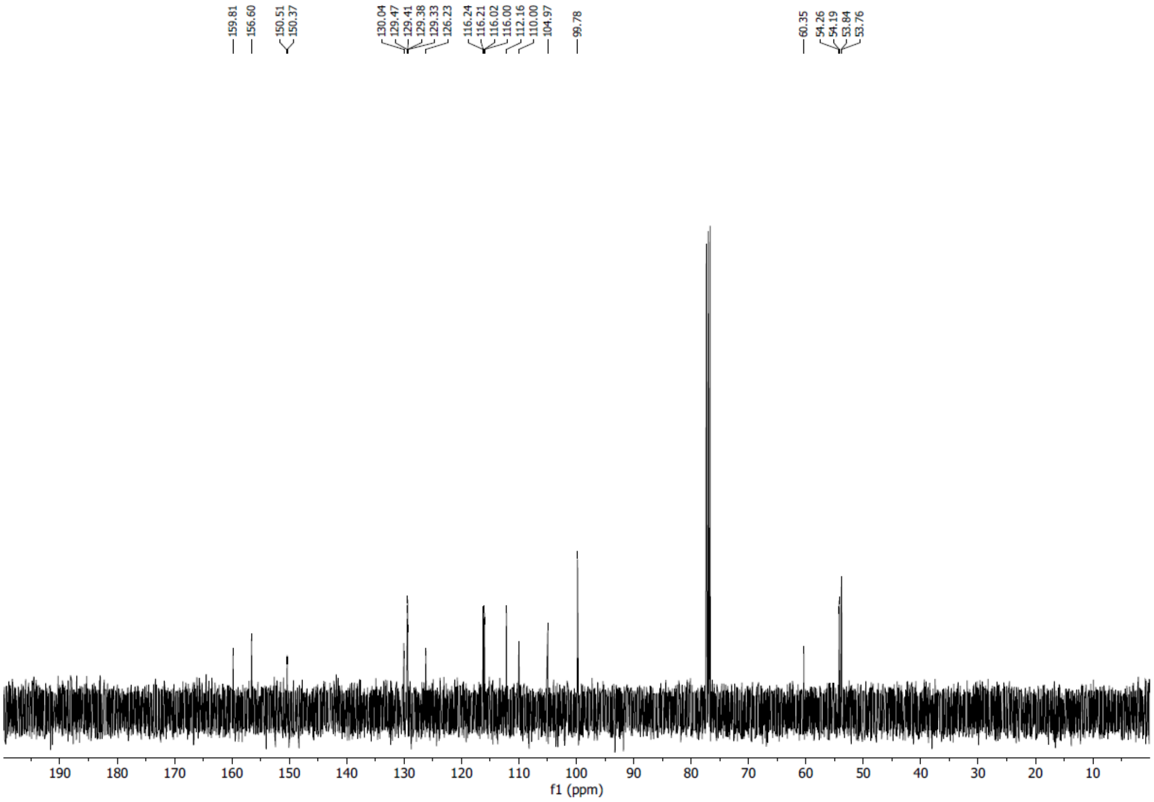

Figure S8. ^{13}H NMR (100 MHz, $CDCl_3$) spectra of compound **4**

Dimethyl ((4-bromophenyl)((2-oxo-4-(trifluoromethyl)-2H-chromen-7-yl)amino) methyl)phosphonate (5). ^{1}H NMR (400 MHz, $CDCl_3$) δ 7.49 (d, *J* = 8.4 Hz, 2H), 7.43 (dd, *J* = 8.9, 1.9 Hz, 1H), 7.37–7.29 (m, 2H), 6.63 (dd, *J* = 8.9, 2.4 Hz, 1H), 6.43 (d, *J* = 8.4 Hz, 2H), 5.84 (t, *J* = 8.8 Hz, 1H), 4.77 (dd, *J* = 24.4, 7.5 Hz, 1H), 3.80 (d, *J* = 10.9 Hz, 3H), 3.53 (d, *J* = 10.7 Hz, 3H); ^{13}C NMR (100 MHz, $CDCl_3$) δ 159.7, 156.5, 150.2, 141.4, 133.4, 132.3, 132.2, 129.3, 126.2, 122.7, 112.1, 105.0, 99.8, 55.4, 54.3, 54.2, 53.9, 53.8.

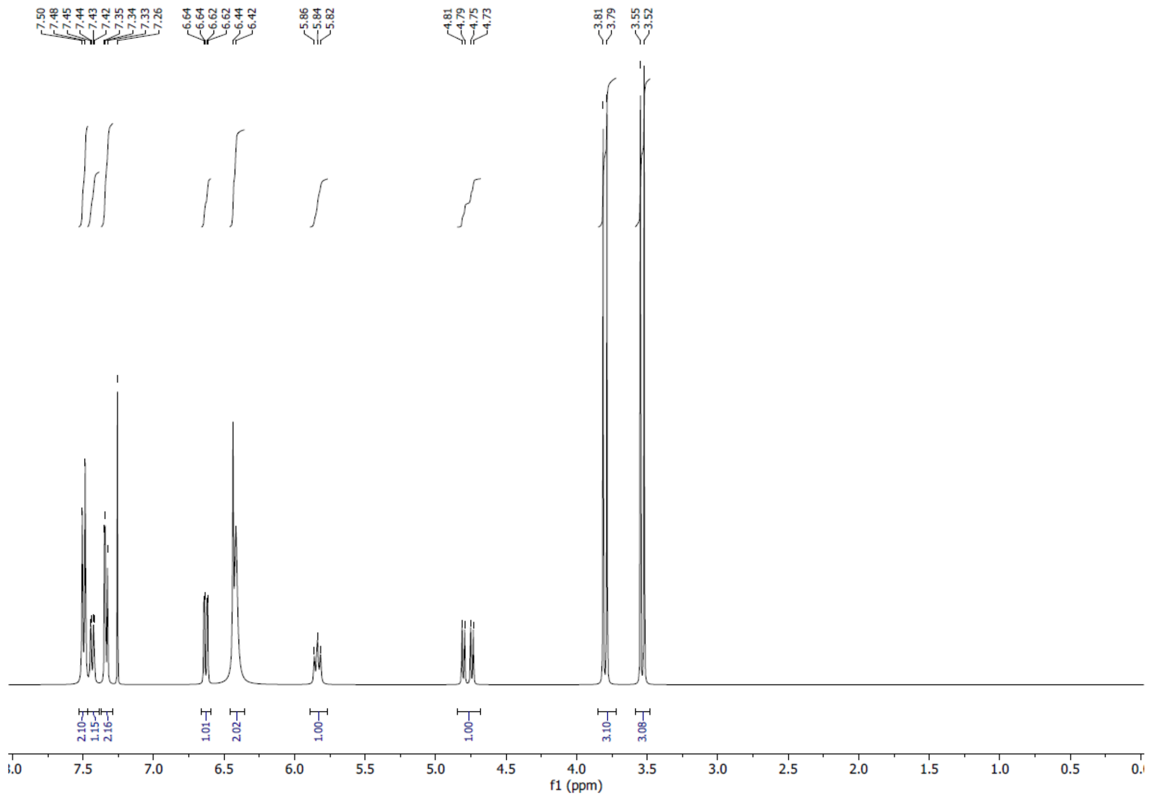

Figure S9. ^{1}H NMR (400 MHz, $CDCl_3$) spectra of compound **5**

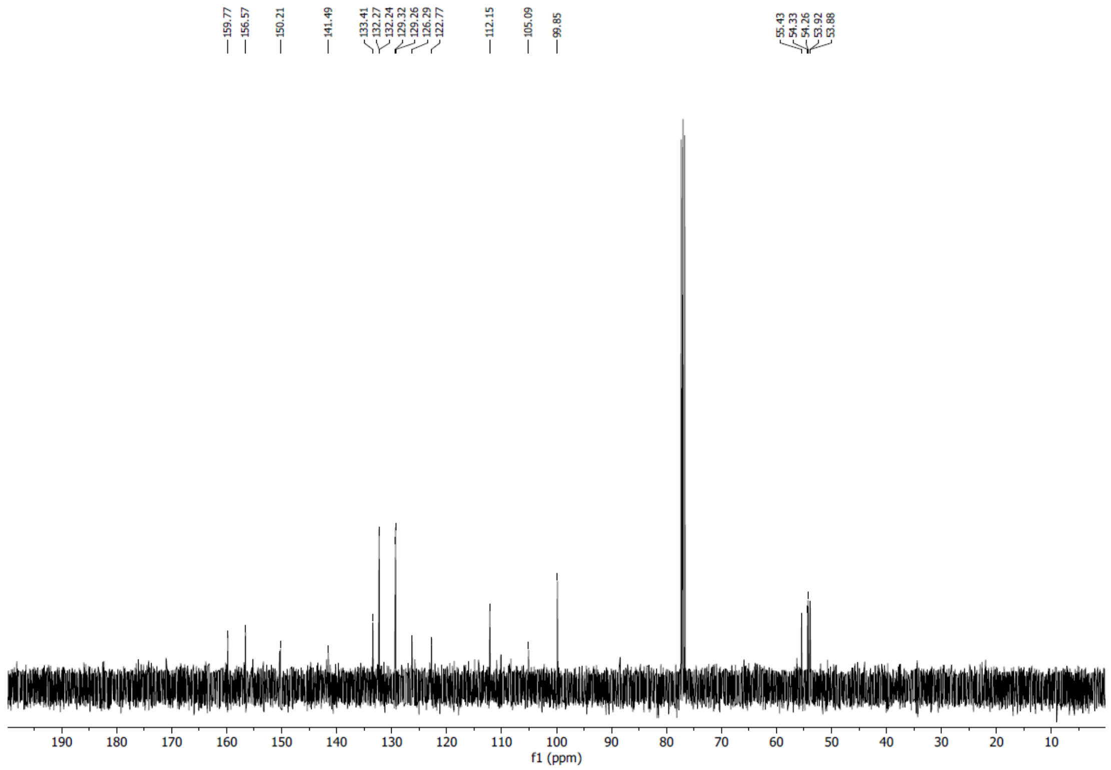

Figure S10. ^{13}H NMR (100 MHz, $CDCl_3$) spectra of compound **5**

Dimethyl ((4-iodophenyl)((2-oxo-4-(trifluoromethyl)-2H-chromen-7-yl)amino) methyl)phosphonate (6). ^{1}H NMR (400 MHz, $CDCl_3$) δ 7.69 (d, J = 7.8 Hz, 2H), 7.42 (dd, J = 8.9, 1.9 Hz, 1H), 7.21 (dd, J = 8.5, 2.3 Hz, 2H), 6.63 (dd, J = 8.9, 2.4 Hz, 1H), 6.42 (d, J = 7.8 Hz, 2H), 5.94 (dd, J = 9.7, 7.7 Hz, 1H), 4.76 (dd, J = 24.4, 7.6 Hz, 1H), 3.80 (d, J = 10.8 Hz, 3H), 3.54 (d, J = 10.7 Hz, 3H); ^{13}C NMR (100 MHz, $CDCl_3$) δ 159.7, 156.5, 150.4, 150.2, 138.1, 134.1, 129.5, 126.2, 123.0, 112.1, 110.0, 105.0, 99.8, 94.3, 55.5, 54.3, 54.0, 53.9, 53.8.

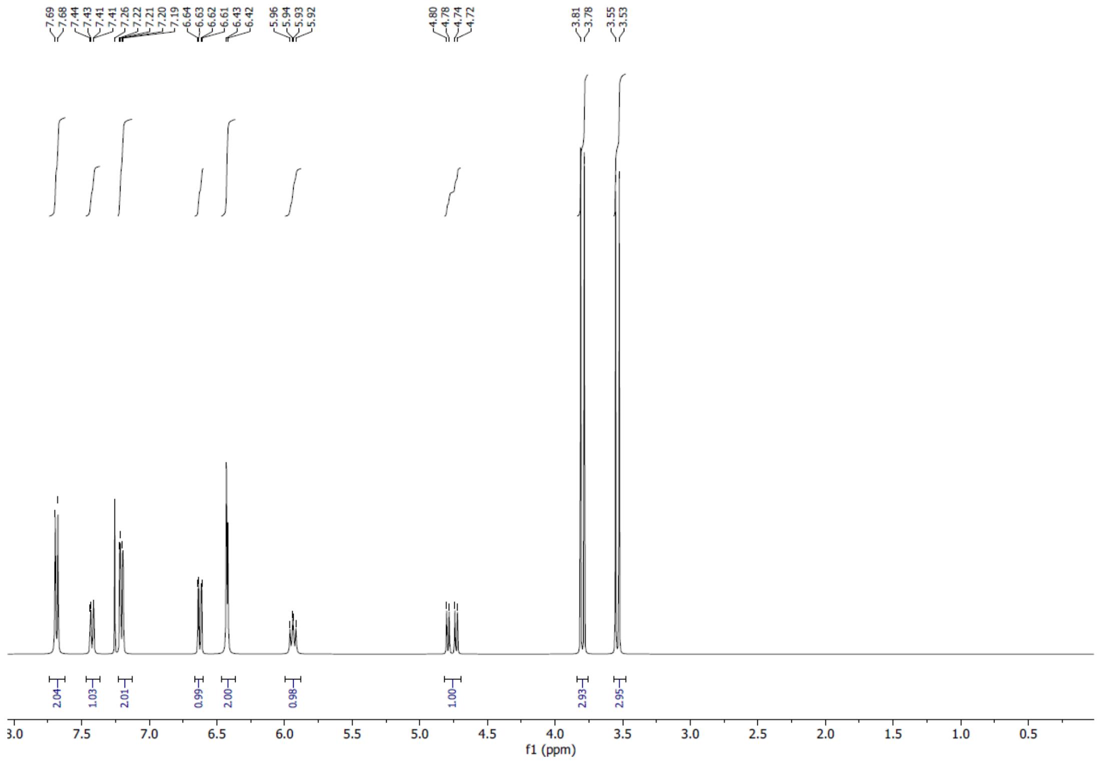

Figure S11. ^{1}H NMR (400 MHz, $CDCl_3$) spectra of compound **6**

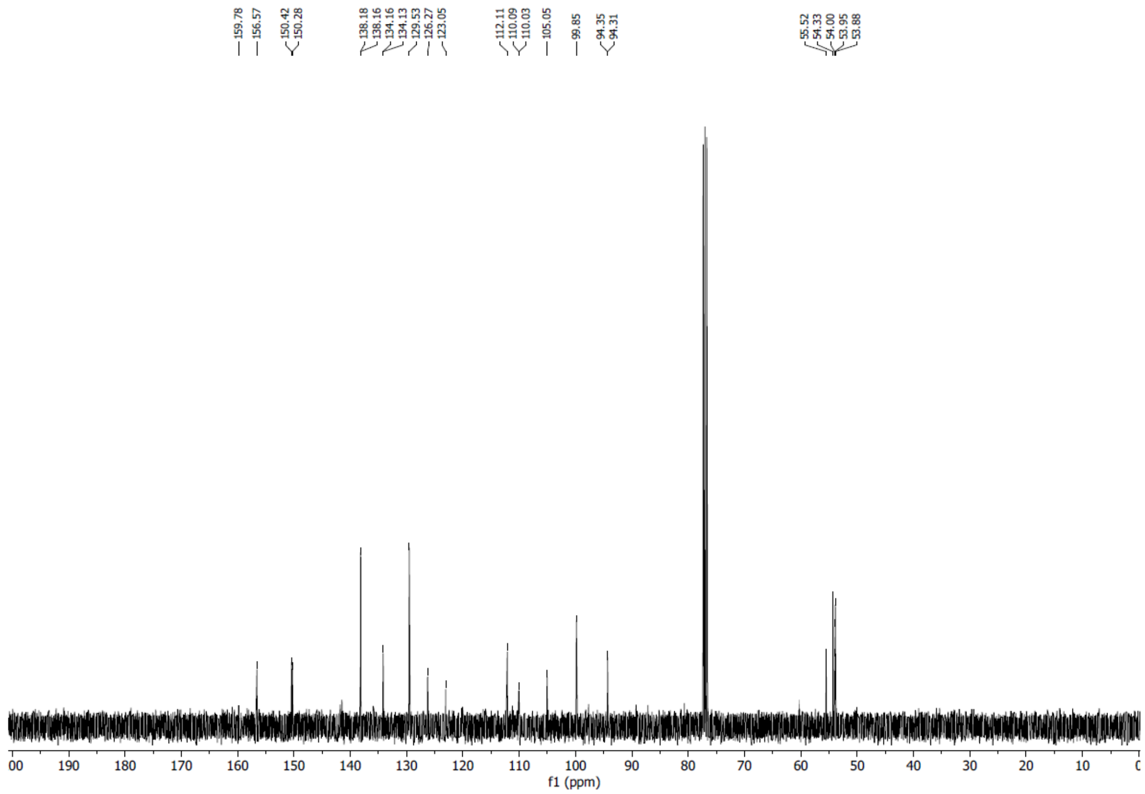

Figure S12. ^{13}H CMR (100 MHz, $CDCl_3$) spectra of compound **6**

Dimethyl ((4-chlorophenyl)((2-oxo-4-(trifluoromethyl)-2H-chromen-7-yl)amino) methyl)phosphonate (7). ^{1}H NMR (400 MHz, $CDCl_3$) δ 7.44–7.39 (m, 2H), 7.34 (d, *J* = 8.7 Hz, 2H), 6.72 – 6.60 (m, 1H), 6.43 (d, *J* = 8.7 Hz, 2H), 5.80 (t, *J* = 8.6 Hz, 1H), 4.79 (dd, *J* = 24.3, 7.5 Hz, 1H), 3.80 (d, *J* = 10.8 Hz, 3H), 3.53 (d, *J* = 10.8 Hz, 3H); ^{13}C NMR (100 MHz, $CDCl_3$) δ 159.7, 156.5, 150.2, 134.6, 132.8, 129.3, 129.2, 129.0, 128.9, 126.2, 112.1, 110.0, 105.0, 99.8, 55.3, 54.3, 53.9, 53.8.

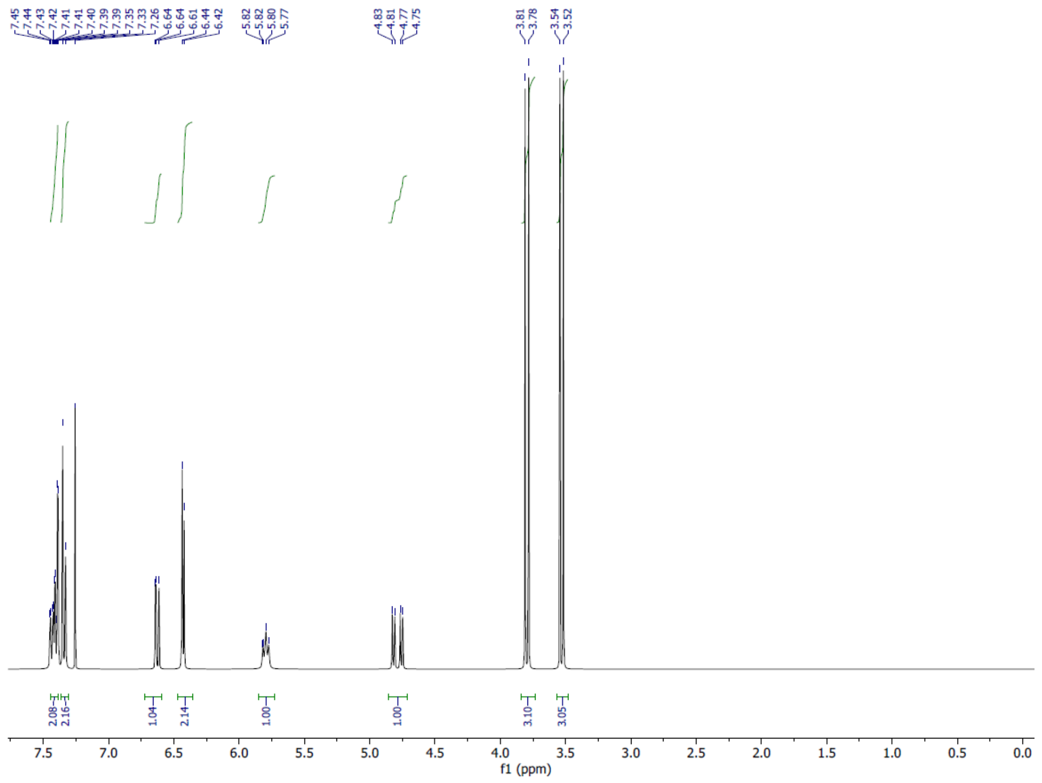

Figure S13. ^{1}H NMR (400 MHz, $CDCl_3$) spectra of compound **7**

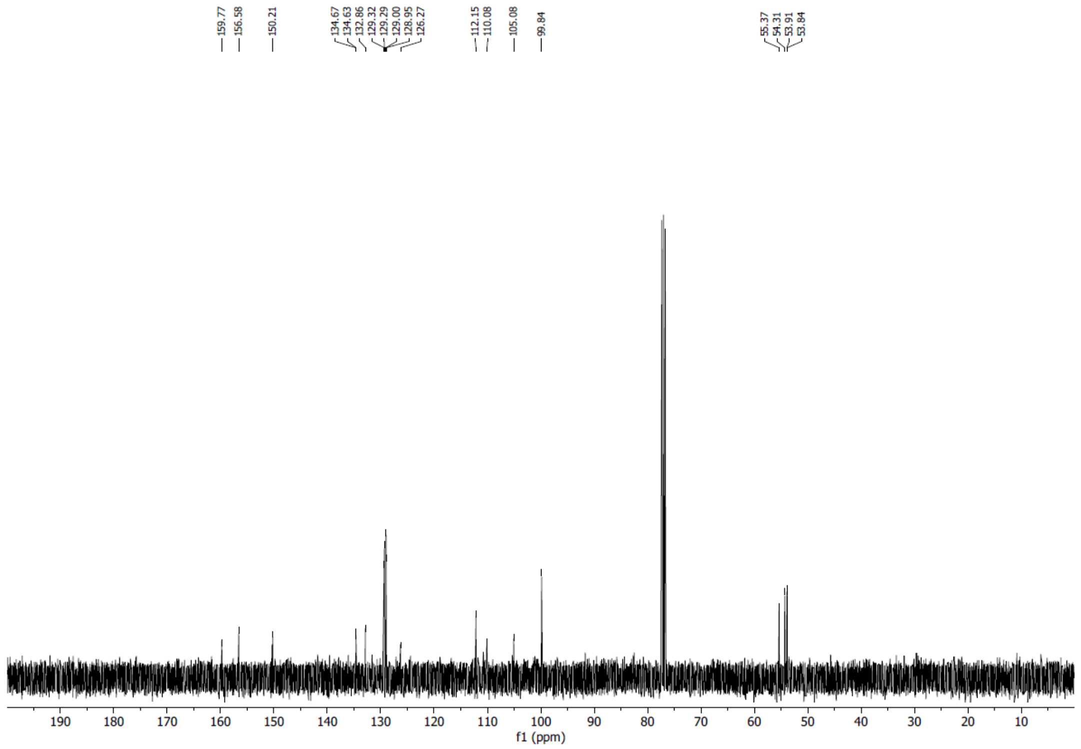

Figure S14. ^{13}H CMR (400 MHz, CDCl3) spectra of compound **7**

Dimethyl ((3-nitrophenyl)((2-oxo-4-(trifluoromethyl)-2H-chromen-7-yl)amino) methyl)phosphonate (8). ^{1}H NMR (400 MHz, $CDCl_3$) δ 8.36 (d, *J* = 2.2 Hz, 1H), 8.16 (dd, *J* = 8.2, 1.0 Hz, 1H), 7.82 (d, *J* = 7.6 Hz, 1H), 7.56 (t, *J* = 8.0 Hz, 1H), 7.43 (dd, *J* = 8.9, 1.9 Hz, 1H), 6.67 (dd, *J* = 8.9, 2.4 Hz, 1H), 6.44 (s, 2H), 6.28 (t, *J* = 8.7 Hz, 1H), 4.98 (dd, *J* = 24.9, 7.7 Hz, 1H), 3.86 (d, *J* = 10.9 Hz, 3H), 3.65 (d, *J* = 10.8 Hz, 3H); ^{13}C NMR (100 MHz, $CDCl_3$) δ 156.5, 150.2, 150.0, 148.7, 137.2, 137.1, 133.8, 133.7, 129.9, 129.9, 126.4, 123.5, 123.5, 122.4, 122.3, 111.9, 105.2, 99.8, 55.3, 54.3, 54.1, 53.8.

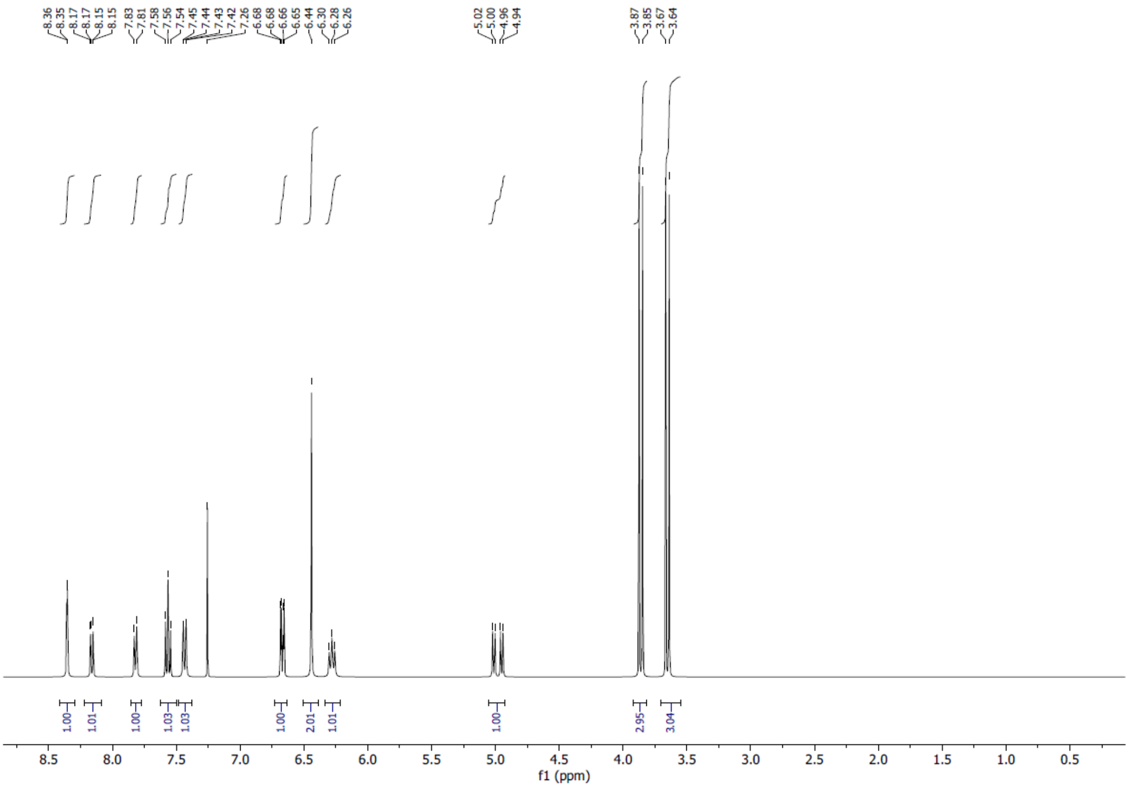

Figure S15. ^{1}H NMR (400 MHz, $CDCl_3$) spectra of compound **8**

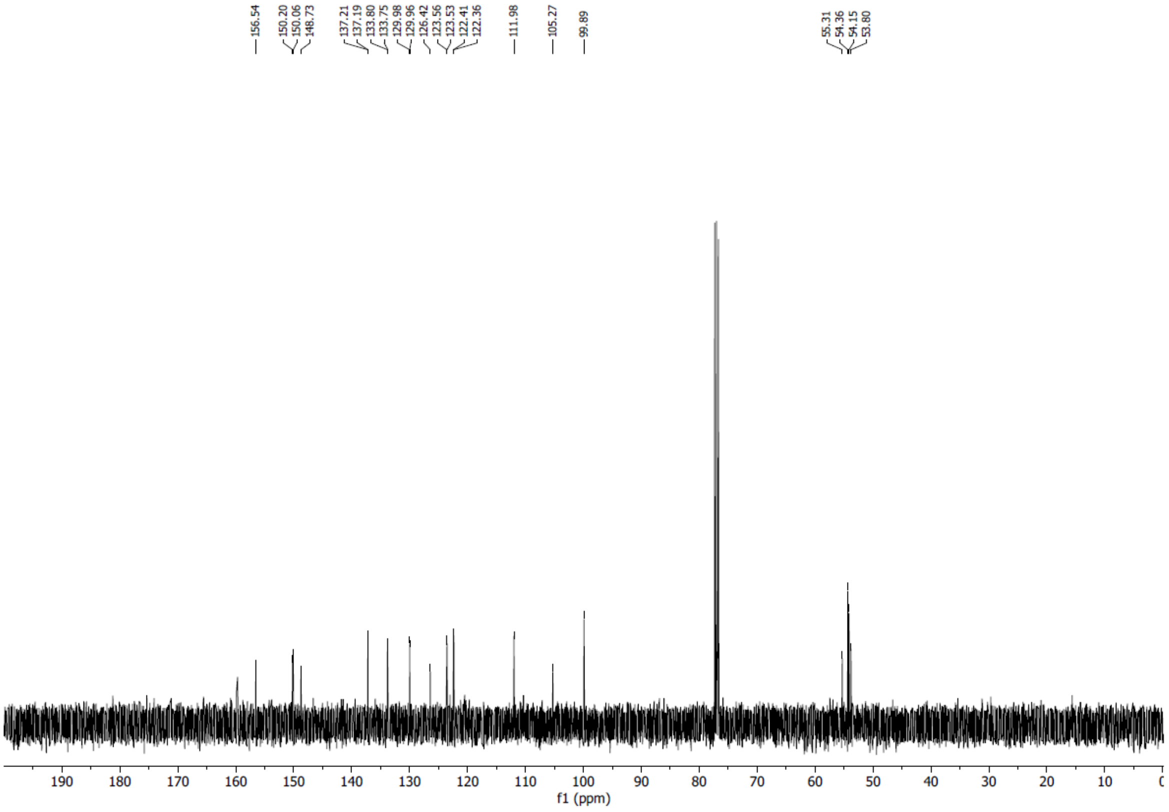

Figure S16. ^{13}H NMR (100 MHz, $CDCl_3$) spectra of compound **8**

Dimethyl ((4-nitrophenyl)((2-oxo-4-(trifluoromethyl)-2H-chromen-7-yl)amino) methyl)phosphonate (9). ^{1}H NMR (400 MHz, $CDCl_3$) δ 8.21 (d, *J* = 8.8 Hz, 2H), 7.66 (d, *J* = 11.0 Hz, 2H), 7.47 – 7.39 (m, 1H), 6.63 (dd, *J* = 8.9, 2.4 Hz, 1H), 6.43 (d, *J* = 8.8 Hz, 2H), 6.01 (dd, *J* = 10.0, 7.4 Hz, 1H), 4.96 (dd, *J* = 25.2, 7.5 Hz, 1H), 3.83 (d, *J* = 10.8 Hz, 3H), 3.62 (d, *J* = 10.8 Hz, 3H); ^{13}C NMR (100 MHz, $CDCl_3$) δ 159.5, 156.5, 150.0, 149.9, 148.0, 142.1, 142.0, 128.6, 128.5, 124.1, 111.9, 105.3, 99.9, 55.6, 54.3, 54.2, 54.1, 54.0.

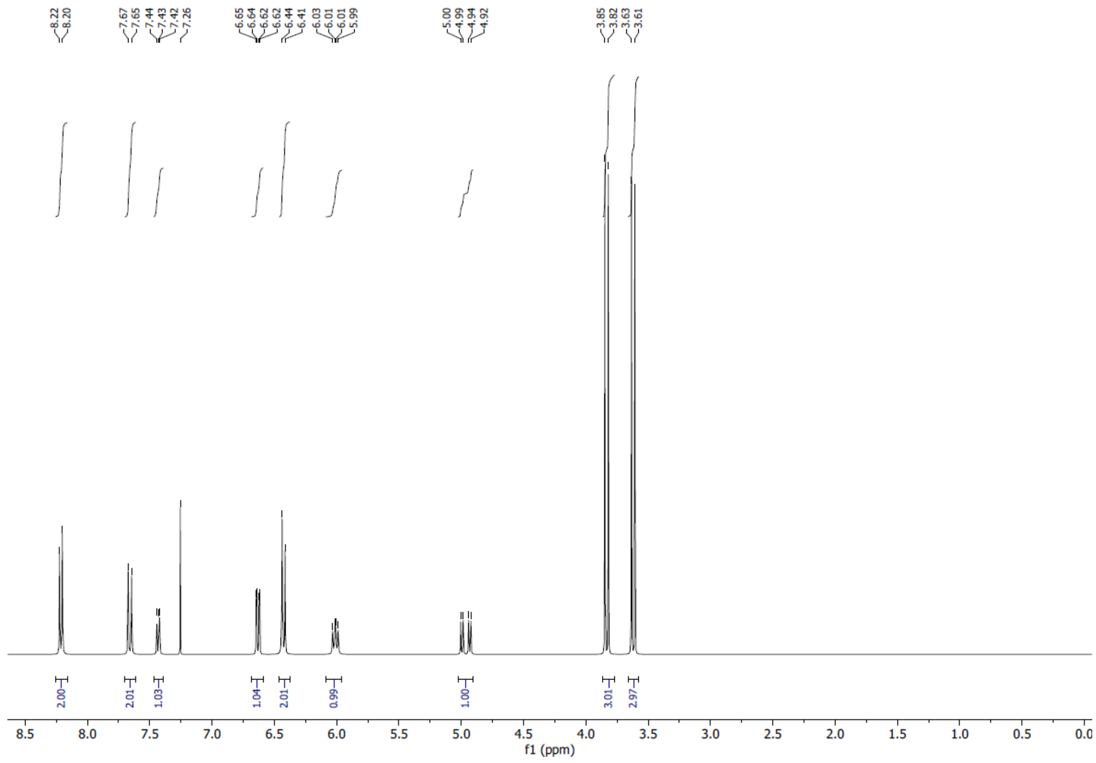

Figure S17. ^{1}H NMR (400 MHz, $CDCl_3$) spectra of compound **9**

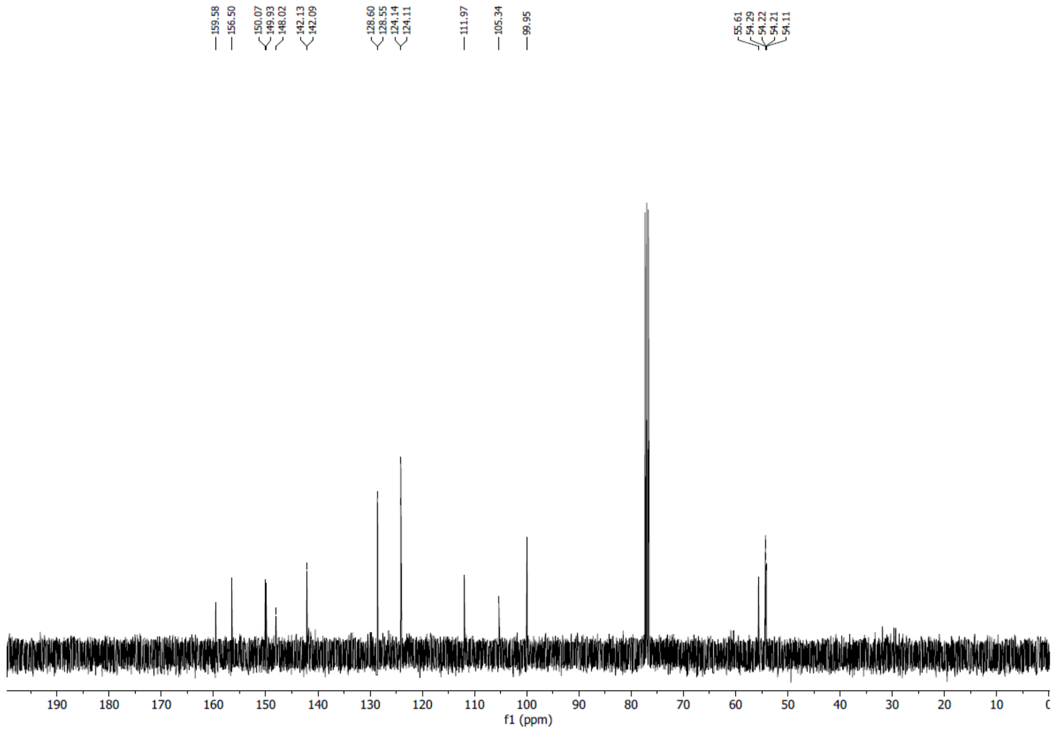

Figure S18. ^{13}H NMR (100 MHz, $CDCl_3$) spectra of compound **9**

Dimethyl ((furan-2-yl)((2-oxo-4-(trifluoromethyl)-2H-chromen-7-yl)amino) methyl)phosphonate (10). ^{1}H NMR (400 MHz, $CDCl_3$) δ 7.50–7.42 (m, 1H), 7.41 (q, J = 1.6 Hz, 1H), 6.67 (dd, J = 8.9, 2.4 Hz, 1H), 6.57 (d, J = 2.4 Hz, 1H), 6.45 (s, 2H), 6.36 (dd, J = 3.4, 1.9 Hz, 1H), 5.52 (t, J = 8.0 Hz, 1H), 4.95 (dd, J = 23.5, 8.7 Hz, 1H), 3.81 (d, J = 10.8 Hz, 3H), 3.63 (d, J = 10.7 Hz, 3H); ^{13}C NMR (100 MHz, $CDCl_3$) δ 159.8, 156.6, 150.3, 150.2, 147.5, 143.1, 126.2, 112.1, 111.0, 110.9, 109.5, 105.1, 99.6, 54.1, 53.9, 53.8, 48.2.

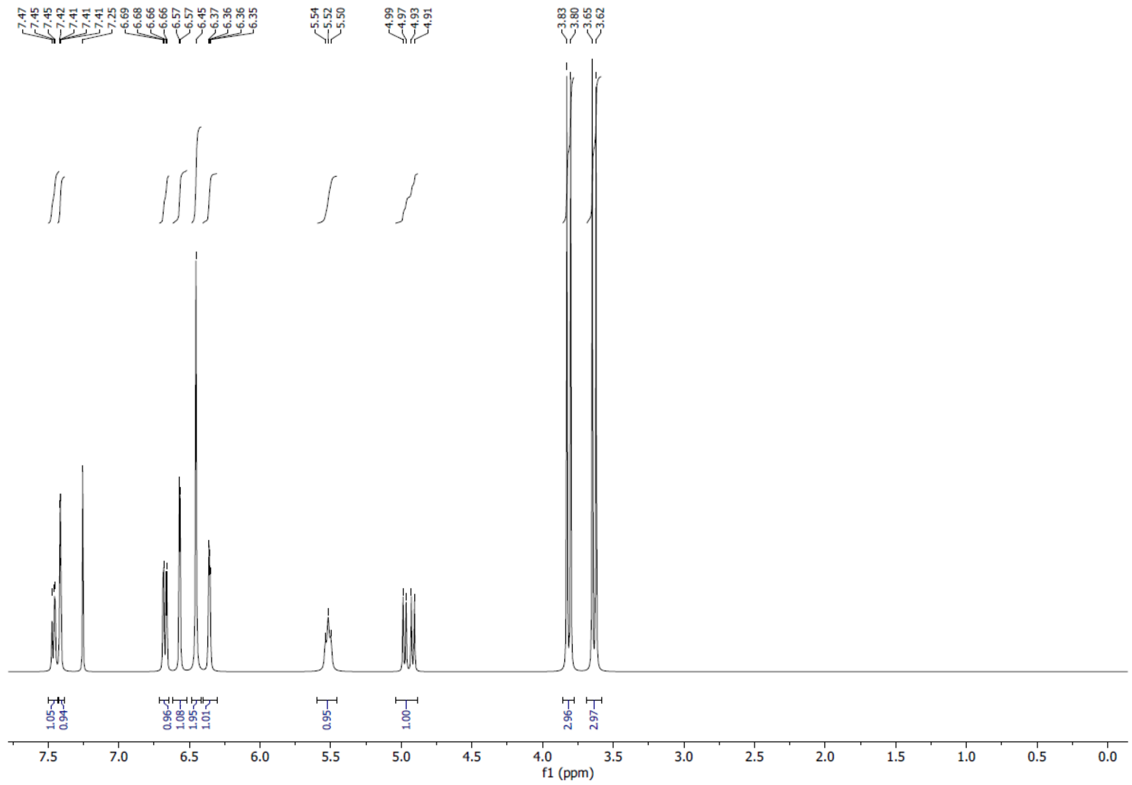

Figure S19. ^{1}H NMR (400 MHz, $CDCl_3$) spectra of compound **10**

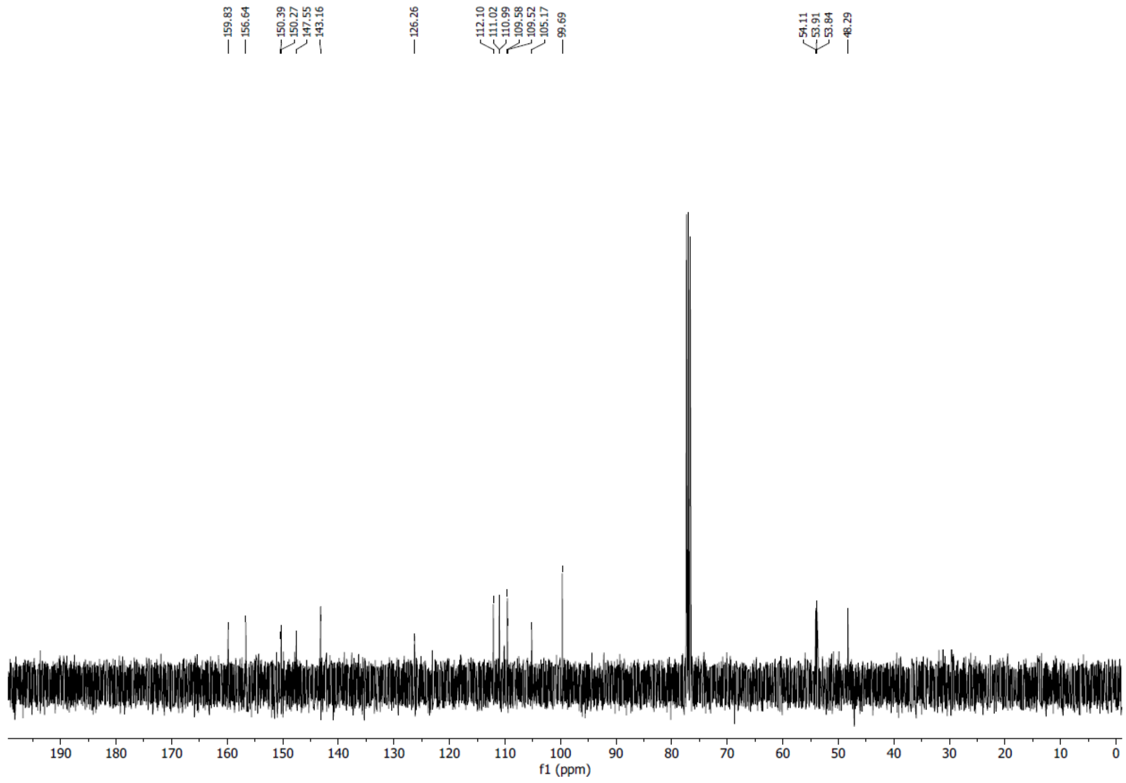

Figure S20. ^{13}H NMR (100 MHz, $CDCl_3$) spectra of compound **10**

Dimethyl ((tiophen-2-yl)((2-oxo-4-(trifluoromethyl)-2H-chromen-7-yl)amino) methyl)phosphonate (11). ^{1}H NMR (400 MHz, $CDCl_3$) δ 7.46 (dd, *J* = 9.0, 1.9 Hz, 1H), 7.30–7.25 (m, 1H), 7.20 (td, *J* = 2.9, 2.4, 1.7 Hz, 1H), 7.00 (ddd, *J* = 5.1, 3.6, 0.7 Hz, 1H), 6.70 (dd, *J* = 8.9, 2.4 Hz, 1H), 6.58 (s, 1H), 6.45 (s, 1H), 5.79 (t, *J* = 8.2 Hz, 1H), 5.11 (dd, *J* = 23.7, 8.0 Hz, 1H), 3.81 (d, *J* = 10.8 Hz, 3H), 3.61 (d, *J* = 10.6 Hz, 3H); ^{13}C NMR (100 MHz, $CDCl_3$) δ 159.8, 156.6, 150.4, 150.3, 137.6, 137.5, 127.4, 127.0, 126.9, 126.2, 126.1, 112.1, 110.1, 110.0, 105.1, 99.8, 54.4, 54.3, 53.9, 51.7, 50.1.

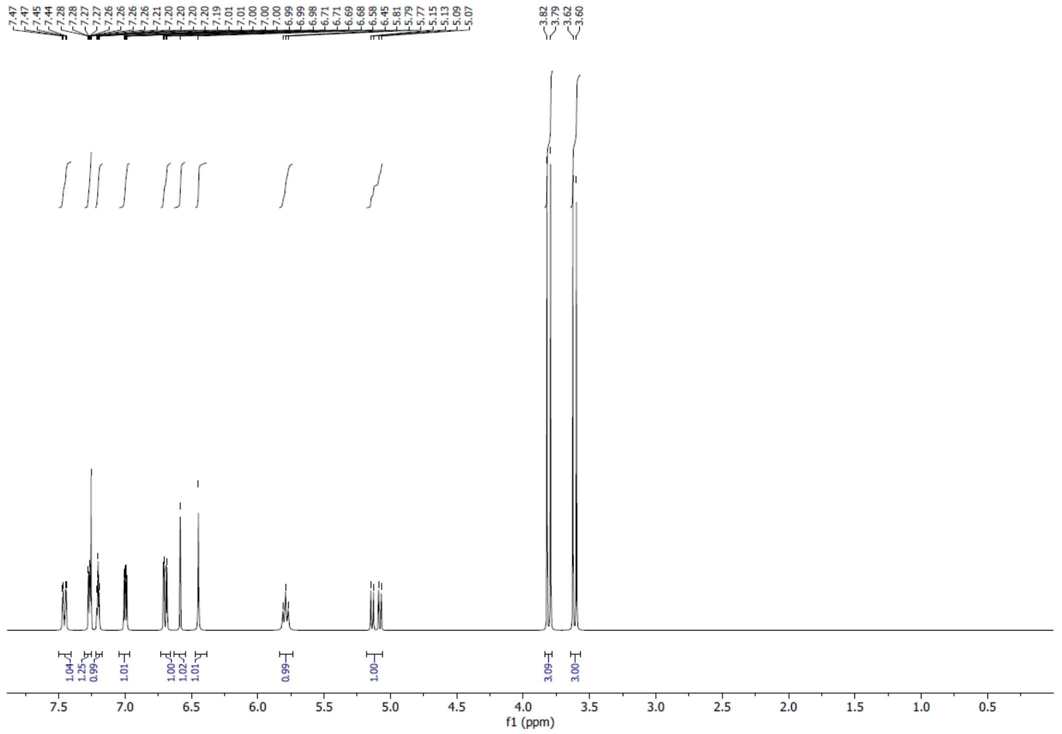

Figure S21. ^{1}H NMR (400 MHz, $CDCl_3$) spectra of compound **11**

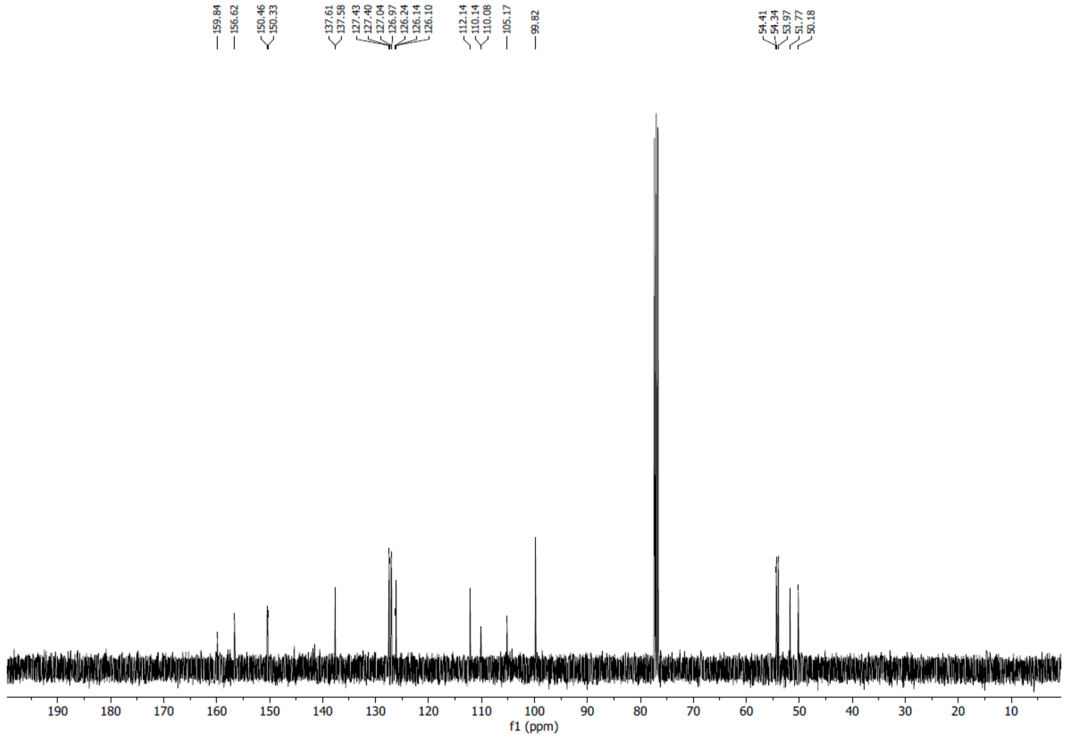

Figure S22. [13]H CMR (100 MHz, $CDCl_3$) spectra of compound **11**

Dimethyl ((naphthalen-1-yl)((2-oxo-4-(trifluoromethyl)-2H-chromen-7-yl)amino) methyl)phosphonate (12). ^{1}H NMR (400 MHz, $CDCl_3$) δ 7.97 (s, 1H), 7.86–7.69 (m, 3H), 7.62 (d, *J* = 8.6 Hz, 1H), 7.49–7.39 (m, 2H), 7.35 (dd, *J* = 8.9, 2.0 Hz, 1H), 6.76–6.66 (m, 1H), 6.61 (t, *J* = 8.6 Hz, 1H), 6.55 (s, 1H), 6.37 (s, 1H), 5.05 (dd, *J* = 24.3, 7.9 Hz, 1H), 3.85 (d, *J* = 10.9 Hz, 3H), 3.48 (d, *J* = 10.6 Hz, 3H); ^{13}C NMR (100 MHz, $CDCl_3$) δ 159.9, 156.5, 151.0, 150.9, 141.7, 141.4, 133.2, 131.8, 128.9, 128.9, 127.9, 127.7, 127.1, 127.0, 126.5, 126.0, 125.2, 125.1, 123.1, 120.3, 112.1, 109.5, 109.5, 104.6, 99.8, 56.0, 54.5, 54.3, 54.2, 53.8.

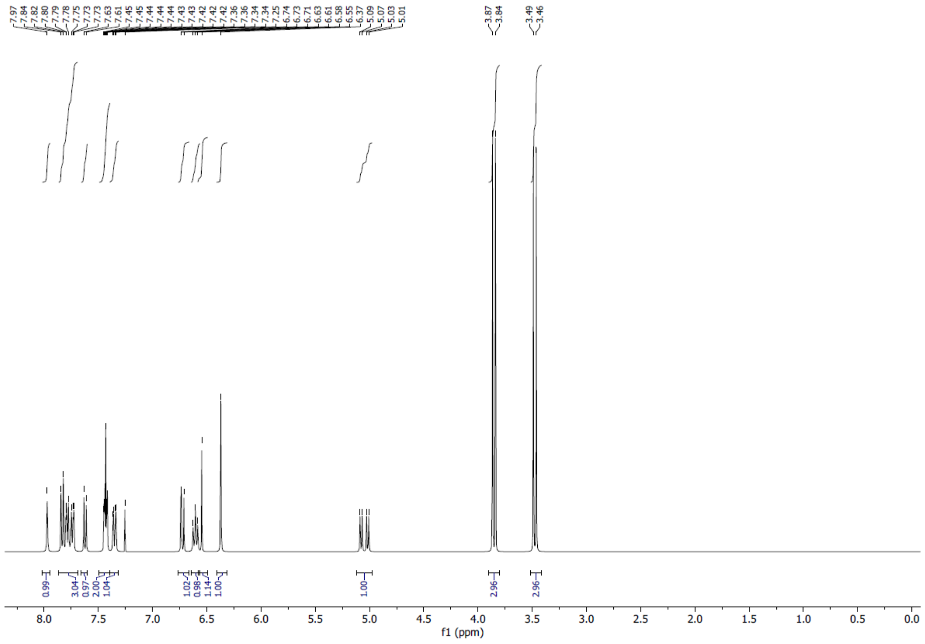

Figure S23. ^{1}H NMR (400 MHz, $CDCl_3$) spectra of compound **12**

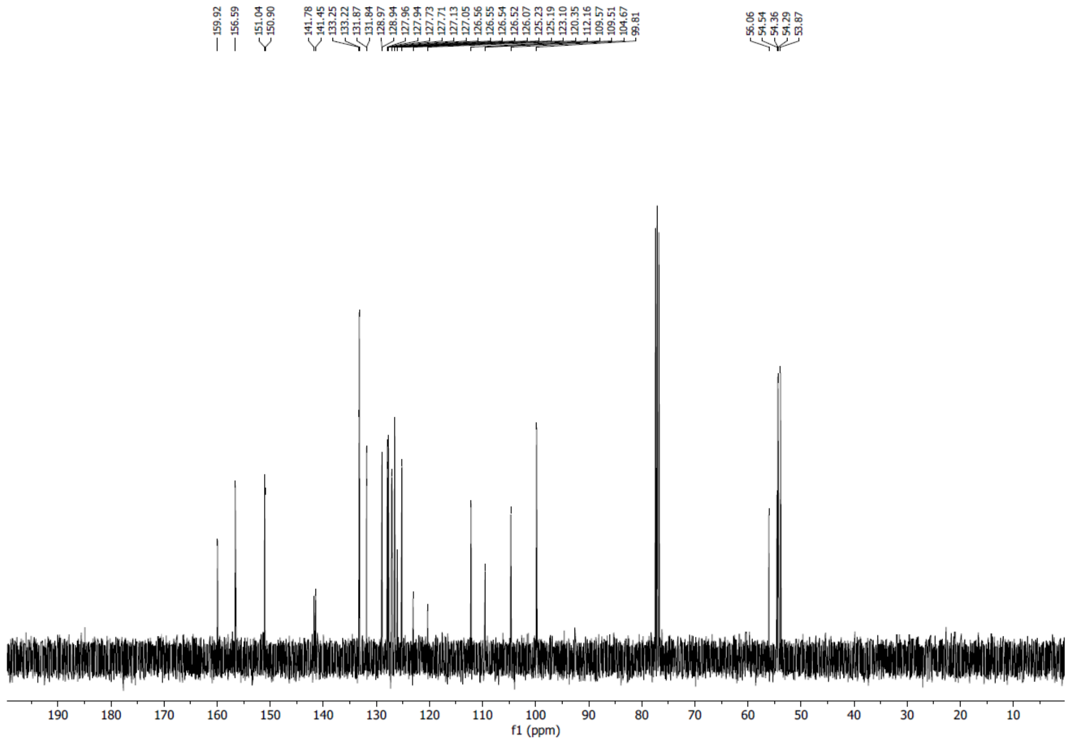

Figure S24. ^{13}H NMR (100 MHz, $CDCl_3$) spectra of compound **12**

Dimethyl ((phenyl)((2-oxo-4-(trifluoromethyl)-2H-chromen-7-yl)amino)methyl) phosphonate (13). ^{1}H NMR (400 MHz, CDCl3) δ 7.49–7.40 (m, 2H), 7.39–7.27 (m, 4H), 6.58 (dd, *J* = 8.7, 2.4 Hz, 1H), 6.40 (s, 1H), 5.95 (s, 1H), 5.49 (dd, *J* = 9.9, 7.7 Hz, 1H), 4.80 (dd, *J* = 24.2, 7.7 Hz, 1H); ^{13}C NMR (100 MHz, $CDCl_3$) δ 161.5, 155.5, 152.6, 149.6, 149.5, 134.5, 128.9, 128.5, 128.4, 127.7, 125.4, 111.6, 111.3, 110.2, 99.7, 56.0, 54.5, 53.7, 53.6, 18.4.

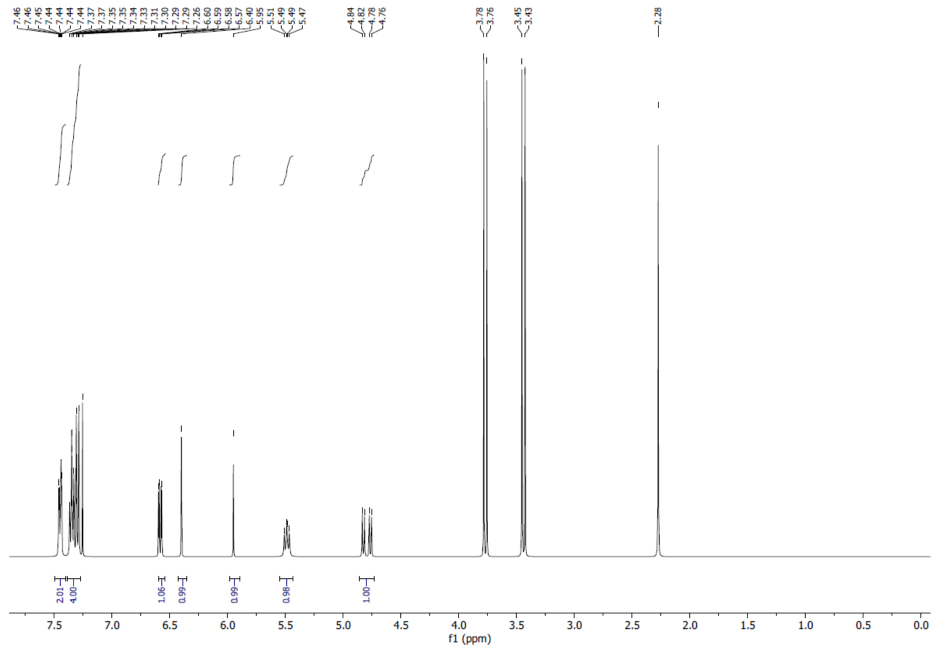

Figure S25. ^{1}H NMR (400 MHz, $CDCl_3$) spectra of compound **13**

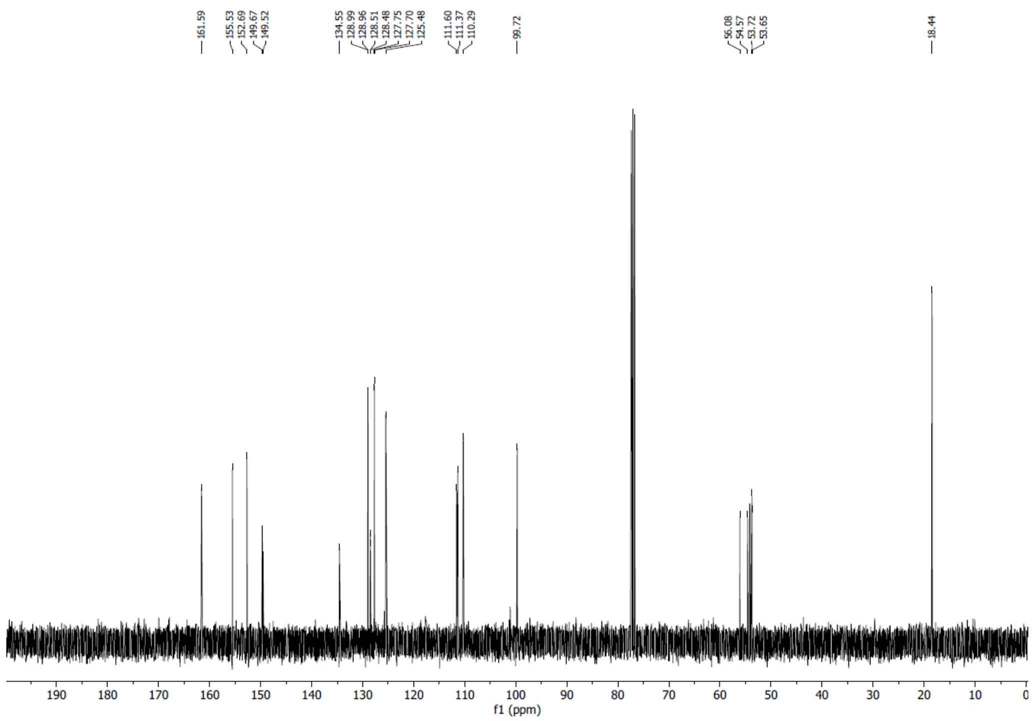

Figure S26. ^{13}H NMR (100 MHz, $CDCl_3$) spectra of compound **13**

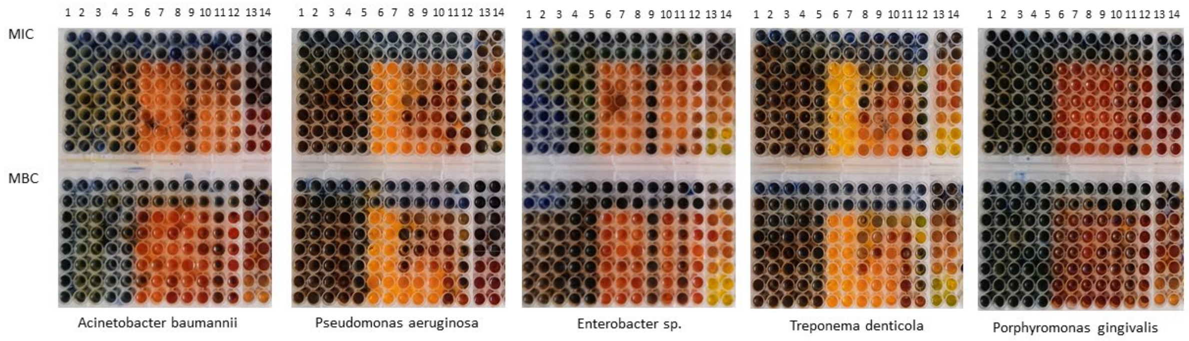

Figure S27. Examples of MIC and MBC on microplates with different concentration of studied compounds (μg/mL^{-1}). Resazurin was added as an indicator of microbial growth with pathogenic analysed strains of with tested compounds.
